# Dynamic performance of functionally graded composite structures with viscoelastic polymers

**DOI:** 10.1038/s41598-024-58399-8

**Published:** 2024-03-31

**Authors:** Shaoqing Wang, Yaqin Song, Yanmei Qiao, Siyuan Shao, Weigang Wang

**Affiliations:** 1 School of Mechanical and Automotive Engineering, Liaocheng University, Liaocheng, 252000 China; 2 School of Aerospace Engineering, Xi’an Jiaotong University, Xi’an, China; 3 Department of Electrical Engineering, Technician College of Liaocheng City, Liaocheng, China; 4 Wendeng Maxpower Tool Group, Weihai, China

**Keywords:** Modeling, Viscoelastic properties, Structure–property relations, Simulations, Structural materials, Theory and computation

## Abstract

The functionally graded composite structures with viscoelastic polymers inherits the excellent performance of functionally graded composites and also possesses large damping performance, which has broad application prospects in the aerospace and mechanical engineering fields. However, due to the complexity of the structure itself, there is limited literature available on its theoretical modeling for efficient solutions. To predict its dynamic performance, a simplified dynamic model of the functionally graded composite structures with viscoelastic polymers is established. This model takes into account the displacement transfer relationship between the functional graded composite layer and the viscoelastic polymer layer. The governing differential equations are obtained by applying the Navier method and complex modulus theory. These equations are then solved using the Rayleigh–Ritz method. The validity of the theoretical model is confirmed by comparing it with existing literature and the results obtained from ANSYS software. Additionally, the model that has been developed is used to analyze how the graded index and elastic modulus of the structure, as well as its geometric parameters, affect its vibration and damping characteristics.

## Introduction

Functionally graded composite materials (FGCMs) are a unique class of composite materials that consist of multiple materials and display a gradual variation in composition throughout space^1–3^. The main objective of FGCMs is to attain diverse properties and functionalities at different locations through precise control of the composition graded. This meticulous control enables a seamless transition from one material to another, thereby minimizing stress concentrations and bolstering the load-bearing capacity of functionally graded composite structures^4,5^. Consequently, these structures exhibit enhanced performance and durability.

In recent years, researchers have conducted significant research on functionally graded composite materials due to their numerous benefits. This research has focused on various aspects, including specimen preparation, prediction of mechanical properties, as well as optimization and enhancement of these properties^6,7^. Currently, there are several methods available for the preparation of graded materials, such as powder metallurgy, plasma spraying, physical vapor deposition, and additive manufacturing^8–11^. For example, Chauhan et al.^12^ utilized the powder metallurgy process to fabricate an aluminum-copper functionally graded material. They investigated the impact of different preparation parameters on the formation of the material's microstructure. Additionally, they assessed potential defects such as voids, porosity, and cracks, and analyzed their influence on the material's properties. Another study conducted by Sain et al.^13^ focused on the preparation and properties of uniform and functionally graded glass fiber-reinforced polymer composites. By altering the distribution of glass fibers in the composite material, they were able to achieve materials with different properties in different regions. The researchers investigated the effects of different preparation methods, process parameters, and fiber distribution on the mechanical properties, thermal performance, and interface characteristics of the composite materials. Put et al.^14^ studied the material selection, preparation methods, and characterization techniques involved in the fabrication process of graded ceramic–metal composite materials. They also proposed strategies for improving and optimizing the material properties.

In addition to preparing specimens and characterizing their properties, theoretical research plays a crucial role in studying the structural behavior of functionally graded composite materials^15^. The main objective of theoretical research is to establish appropriate mechanics models and numerical calculation methods. These models are solved using variational principles or Rayleigh–Ritz theory to predict stress and strain distributions at different positions, as well as the variation patterns of mechanical properties with graded index. For instance, Zhao et al.^16^ examined the vibration characteristics of FGC double beams at different temperatures and analyzed the influence of temperature on stiffness, damping, and resonance frequency. Akgöz et al.^17^ used mathematical models and numerical methods to investigate how thermal and shear deformations affect the dynamic performance of ceramic–metal functionally graded thick composite microbeams. Kim et al.^18^ employed the variational principle and finite element method to establish a mechanical model for functionally graded plates, considering geometric and material nonlinearity effects. They accurately predicted the mechanical behavior of the plates and validated the method's effectiveness and accuracy through numerical examples and comparative analysis. Atmane et al.^19^ studied the buckling properties of FGC plates under thermal loading. They investigated the effects of temperature and material gradeds on the plates and analyzed the buckling modes and critical temperature. Raza et al.^20^ introduced random variables to describe material uncertainty and used probability statistical methods to analyze the impact of material uncertainty on the vibration characteristics of cracked FGC plates. Benachour et al.^21^ utilized the refined plate theory with four variables to investigate the free vibration of functional graded plates with arbitrary gradeds. Pandey et al.^22^ divided the laminated shell into multiple layers and used different finite element models to describe material property variations. Kapuria et al.^23^ utilized the third-order shear deformation theory to study the static mechanical properties and vibration characteristics of ceramic-based functionally graded beams. Eghtesad et al.^24^ utilized a corrected smoothed particle method to investigate the mechanical response of ceramic–metal functionally graded materials subjected to high-speed impact conditions.

In order to enhance the flexural stiffness and load-bearing capacity of functionally graded composite materials, researchers have developed a functionally gradient composite sandwich structure (FGCSS)^25–27^. Natarajan et al.^28^ established a precise theoretical framework for analyzing the bending and vibration characteristics of FGCSS. Meanwhile, Pandey et al.^29^ delved into the mechanical behavior and performance of sandwich structures fabricated from functionally graded materials. They formulated a mathematical model based on a higher-order layerwise theory to predict the response of FGCSS accurately under diverse loading conditions. Frostig et al.^30^ investigated the nonlinear wrinkling phenomena of FGCSS with functionally graded cores utilizing an extended high-order methodology. Their research primarily focused on exploring the mechanical response of FGCSS subjected to various loading scenarios, taking into account the impact of material properties and geometric imperfections on wrinkling behavior. However, the core layer of the FGCSS is made of lightweight material instead of viscoelastic damping material, resulting in limited vibration damping performance.

In conclusion, researchers have extensively studied the preparation, mechanical properties, and potential applications of functionally graded composite materials and FGCSS. However, these structures have the disadvantage of poor damping performance^31,32^. To address this limitation, researchers have developed functionally graded composite structures with viscoelastic polymers (FGCSVP). This innovative approach effectively tackles the issue by integrating viscoelastic polymers into the structure. As a result, the FGCSVP is capable of efficiently absorbing and dispersing vibrational energy. This leads to a significant reduction in vibration amplitude, and minimized dynamic response and vibration noise. The FGCSVP not only inherits the excellent performance of FGCMs but also demonstrates outstanding damping properties. So, the structure has broad application prospects in aerospace field, automotive industry, medical structure and other fields, such as aero-engine parts, automotive chassis components, artificial joints, and medical instruments. However, the panel layers in this structure are comprised of functionally graded composites, leading to more intricate constitutive relationships compared to isotropic materials. Yang et al.^33^ combined Fourier series with the Hamiltonian principle to derive a system of controlled differential equations involving nine unknown coefficients. The paper primarily investigated the impact of boundary conditions on the structural vibration characteristics when the graded indices of the upper and lower panels are identical. Nevertheless, the effects of elastic modulus, damping layer location, and aspect ratio on the dynamic behavior of FGCSVP have not been addressed. In the present study, a simplified dynamic model is established based on Rayleigh–Ritz theory to improve computational efficiency. The controlled differential equations generated by this model have only five unknown coefficients, which is four fewer than the unknown coefficients in the control differential equations in reference^33^. The accuracy of the dynamic model is verified through published literature and ANSYS software. Furthermore, the influences of various structural parameters, such as elastic modulus, graded index, viscoelastic polymer layer position, aspect ratio, and layer thickness ratio, on the vibration and damping characteristics of the structure are analyzed and discussed under conditions where the graded indices of the upper and lower panels are either identical or different.

## Vibration equation

### Assumption and material property

To derive the governing equations, we have made several assumptions. Firstly, we neglect deformation in the thickness direction. Secondly, we disregard interlayer interface slip. Thirdly, we assume linear elastic material behavior for the FGCMs layers, where the stress–strain relationship follows Hooke's law. Finally, it is important to note that the elastic parameters of the viscoelastic polymer layer exist in the form of complex modulus.

**Figure 1:**
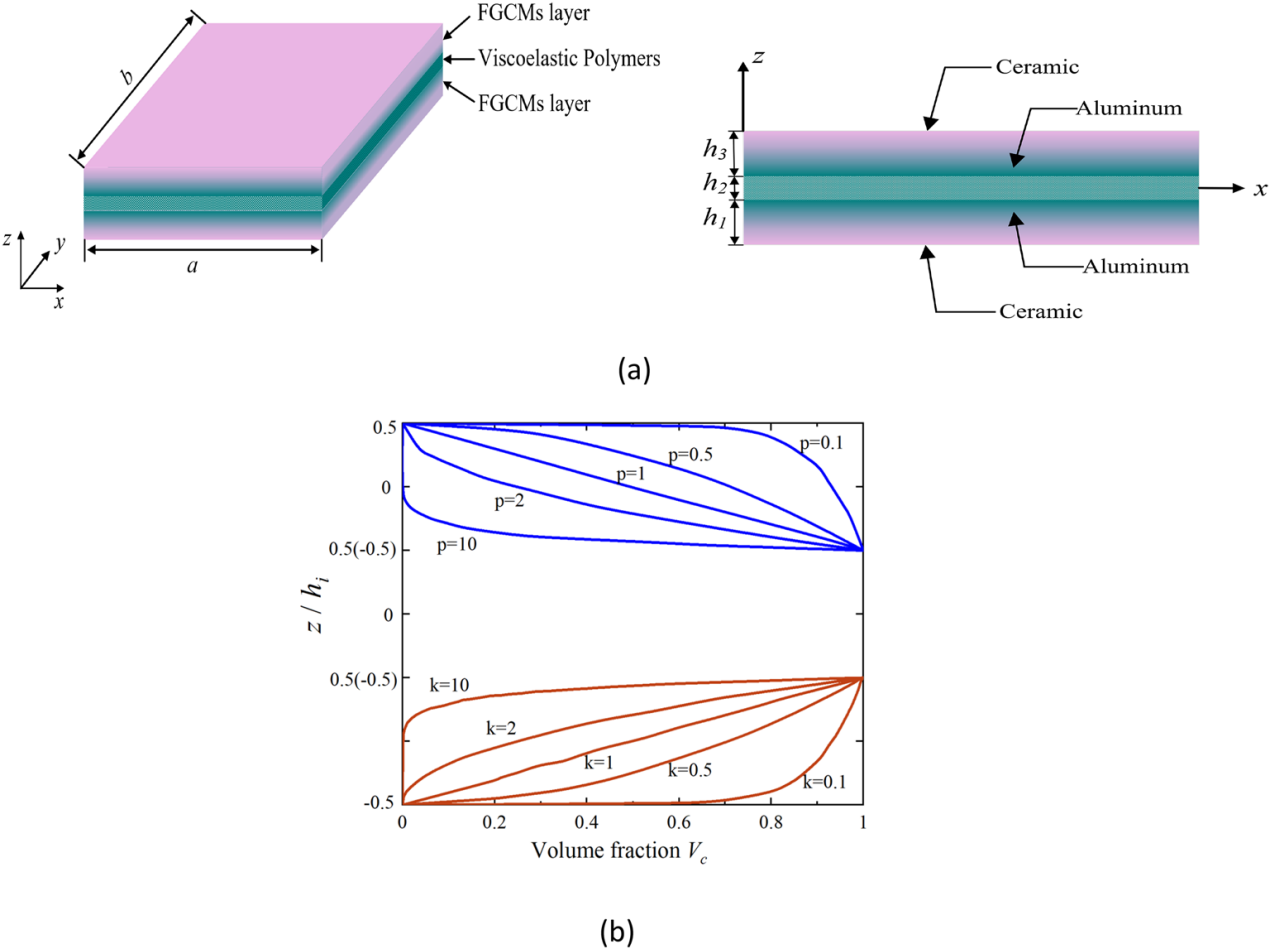
(**a**) Geometric model of FGCSVP; (**b**) variation of volume fraction *V*_*c*_ with plate thickness under different power-law indices *p* and *k.*

Figure 1 depicts the geometric model of FGCSVP and demonstrates variation of volume fraction *V*_*c*_ with plate thickness under different power-law indices *p* and *k*. In this model, *h*_*1*_ and *h*_*3*_ represent the height of the functional graded composite layer, while *h*_*2*_ denotes the height of the viscoelastic polymer layer.

The functional graded composite layer typically consists of two materials: ceramic and metal in Fig. 1a. Ceramic is a non-metallic inorganic material known for its high hardness, wear resistance, and ability to withstand high temperatures. On the other hand, metals possess excellent electrical conductivity, thermal conductivity, and plasticity. By combining ceramic and metal, the benefits of both materials can be fully leveraged, resulting in a composite material with versatile properties. In the context of the functional graded composite layer, we make an assumption that the material properties change gradually and consistently throughout the thickness. To describe these properties, we can use the following expression:

1 $$ P\left( z \right) = \left( {P_{1} - P_{2} } \right)\left( {\frac{z}{{h_{1} }} + 0.5} \right)^{p}  + P_{2}\qquad z \in \left[ { - \frac{{h_{1} }}{2},  \frac{{h_{1} }}{2}} \right] $$

2 $$ P\left( z \right) = \left( {P_{1} - P_{2} } \right)\left( { - \frac{z}{{h_{3} }} + 0.5} \right)^{p}  + P_{2}\qquad z \in \left[ { - \frac{{h_{3} }}{2},  \frac{{h_{3} }}{2}} \right] $$

where $P$ indicates the material properties of constituents, which include Young’s modulus $E(z)$ and density $\rho (z)$; *P*_*1*_ and *P*_*2*_ represent the material properties of two different types of materials, respectively.

The viscoelastic polymers have a high capacity for generating internal friction. When exposed to vibration and noise, the molecular chains in these polymers experience frictional movement, which generates heat. This heat effectively absorbs the energy from the vibration and noise, converting it into heat and dissipating it. As a result, it reduces the transmission of vibration and noise, ultimately achieving the desired outcome of reducing vibration and noise. The elastic parameters of the viscoelastic polymer layer can be described using the constant complex modulus.

3 $$ E_{{}}^{(\nu )} = E_{{}}^{*} (1 + i\eta_{v} ) $$

4 $$ G_{{}}^{(\nu )} = G_{{}}^{*} (1 + i\eta_{{\text{v}}} ) $$

where $E_{{}}^{(\nu )}$ denotes the complex elastic modulus of the viscoelastic polymer; $E_{{}}^{*}$ denotes the storage elastic modulus of the viscoelastic polymer; $G_{{}}^{(\nu )}$ denotes the complex shear modulus of the viscoelastic polymer; $G_{{}}^{*}$ denotes the storage shear modulus of the viscoelastic polymer; and $\eta_{v}^{{}}$ indicates the loss factor of the viscoelastic polymer.

### Constitutive relationship

The displacements, denoted as $\overline{{U_{i} }}    ,  \overline{{V_{{i   }} }} ,\overline{W}$, at any point within the functional graded composite layers can be expressed in terms of their components along the x, y, and z directions as follows:

5a $$ \overline{U}_{i} (x,y,z,t) = u_{i} (x,y,t) - z^{(i)} \frac{\partial w}{{\partial x}} $$

5b $$ \overline{V}_{i} (x,y,z,t) = v_{i} (x,y,t) - z^{(i)} \frac{\partial w}{{\partial y}} $$

5c $$ \overline{W} (x,y,z,t) = w(x,y,t) $$

5d $$ { - }\frac{{h_{i} }}{2} \le z^{(i)} \le \frac{{h_{i} }}{2},   i = 1,  3 $$

where *u*_*i*_, *v*_*i*_, and *w* represent the displacement of the middle plane of the functional graded composite layers along the x, y, and z axes, respectively.

The displacements along the *x*, *y*, and *z* directions at any point in the viscoelastic polymer layer are denoted as $\overline{{U_{2} }}    ,  \overline{{V_{{2   }} }} ,\overline{W}$, respectively.

6a $$ \overline{U}_{2} (x,y,z,t) = u_{2} (x,y,t) + z^{(2)} \alpha_{2} $$

6b $$ \overline{V}_{2} (x,y,z,t) = v_{2} (x,y,t) + z^{(2)} \beta_{2} $$

6c $$ \overline{W} (x,y,z,t) = w(x,y,t) $$

where *u*_*2*_, *v*_*2*_ represent the displacement of the middle plane of the viscoelastic polymer layer, respectively. $\alpha_{2}$ and $\beta_{2}$ are the angles of the normal of the viscoelastic layer with respect to the x and y axes, respectively.

Based on the continuity of interlaminar displacement, we establish the relationship between the functional graded composite layers and the viscoelastic polymer layer as follows:

7a $$ u_{2} = \frac{1}{2}\left( {u_{1} + u_{3} } \right) + \frac{1}{4}\left( {h_{3} - h_{1} } \right)\frac{\partial w}{{\partial x}} $$

7b $$ v_{2} = \frac{1}{2}\left( {v_{1} + v_{3} } \right) + \frac{1}{4}\left( {h_{3} - h_{1} } \right)\frac{\partial w}{{\partial y}} $$

7c $$ \alpha_{2} = \frac{1}{{h_{2} }}\left( {u_{3} - u_{1} } \right) + \frac{1}{{2h_{2} }}\frac{\partial w}{{\partial x}}\left( {h_{1} + h_{3} } \right) $$

7d $$ \beta_{2} = \frac{1}{{h_{2} }}\left( {v_{3} - v_{1} } \right) + \frac{1}{{2h_{2} }}\frac{\partial w}{{\partial y}}\left( {h_{1} + h_{3} } \right) $$

The strain of the functional graded composite layers and the viscoelastic polymer layer can be described as follows:

8 $$ \left[ \begin{gathered}   \varepsilon _{x}^{{(i)}}  \hfill \\   \varepsilon _{y}^{{(i)}}  \hfill \\   \gamma _{{xy}}^{{(i)}}  \hfill \\   \gamma _{{yz}}^{{(i)}}  \hfill \\   \gamma _{{xz}}^{i}  \hfill \\  \end{gathered}  \right] = \left[ {\begin{array}{*{20}c}    {\partial /\partial x} & 0 & 0  \\    0 & {\partial /\partial y} & 0  \\    {\partial /\partial y} & {\partial /\partial x} & 0  \\    0 & {\partial /\partial z} & {\partial /\partial y}  \\    {\partial /\partial z} & 0 & {\partial /\partial x}  \\   \end{array} } \right]\,{\kern 1pt} \left[ \begin{gathered}   \overline{{U_{i} }}  \hfill \\   \overline{{V_{i} }}  \hfill \\   \overline{{W_{i} }}  \hfill \\  \end{gathered}  \right] $$

The stress distribution within the functional graded composite layers and the rubber layer can be described in the following way:

9a $$ \left[ \begin{gathered}   \sigma _{x}^{{(i)}}  \hfill \\   \sigma _{y}^{{(i)}}  \hfill \\   \tau _{{xy}}^{{(i)}}  \hfill \\  \end{gathered}  \right] = \left[ {\begin{array}{*{20}c}    {\overline{Q} _{{11}}^{{(i)}} } & {\overline{Q} _{{12}}^{{(i)}} } & {\overline{Q} _{{16}}^{{(i)}} }  \\    {\overline{Q} _{{12}}^{{(i)}} } & {\overline{Q} _{{22}}^{{(i)}} } & {{\kern 1pt} \overline{Q} _{{26}}^{{(i)}} }  \\    {\overline{Q} _{{16}}^{{(i)}} } & {\overline{Q} _{{26}}^{{(i)}} } & {\overline{Q} _{{66}}^{{(i)}} }  \\   \end{array} } \right]\left[ \begin{gathered}   \varepsilon _{x}^{{(i)}}  \hfill \\   \varepsilon _{y}^{{(i)}}  \hfill \\   \gamma _{{xy}}^{{(i)}}  \hfill \\  \end{gathered}  \right] $$

9b $$ \left[ \begin{gathered}   \tau _{{yz}}^{{(i)}}  \hfill \\   \tau _{{xz}}^{{(i)}}  \hfill \\  \end{gathered}  \right] = \left[ {\begin{array}{*{20}c}    {\overline{Q} _{{44}}^{{(i)}} } & {\overline{Q} _{{45}}^{{(i)}} }  \\    {\overline{Q} _{{45}}^{{(i)}} } & {\overline{Q} _{{55}}^{{(i)}} }  \\   \end{array} } \right]\left[ \begin{gathered}   \gamma _{{yz}}^{{(i)}}  \hfill \\   \gamma _{{xz}}^{{(i)}}  \hfill \\  \end{gathered}  \right] $$

### Derivation of governing equations

To accurately capture the complex dynamics of the structure, the strain and kinetic energies of the FGCSVP are formulated separately.

10 $$ U = \frac{1}{2}\sum\limits_{i = 1}^{3} {\iiint\limits_{V} {\left( {\varepsilon_{x}^{(i)} \sigma_{x}^{(i)} + \varepsilon_{y}^{(i)} \sigma_{y}^{(i)} + \gamma_{xy}^{(i)} \tau_{xy}^{(i)} + \gamma_{yz}^{(i)} \tau_{yz}^{(i)} + \gamma_{xz}^{(i)} \tau_{xz}^{(i)} } \right)}} \,dV $$

11 $$ T = \frac{1}{2}\iiint {\left[ {\sum\limits_{i = 1}^{3} {\rho_{i} \left( {\frac{{\partial \overline{U}_{i} }}{\partial t}} \right)^{2} + \sum\limits_{i = 1}^{3} {\rho_{i} \left( {\frac{{\partial \overline{V}_{i} }}{\partial t}} \right)^{2} + \rho \left( {\frac{\partial w}{{\partial t}}} \right)^{2} } } } \right]}dV $$

where the variable $\rho_{i}$ represents the density of the ith layer, while the variable $\rho$ represents the density of the FGCSVP.

The boundary conditions of FGCSVP are defined as simply supported on all four sides. In accordance with Navier's procedure for addressing the vibration displacement equation, the displacement parameters were represented using Fourier series, as demonstrated below.

12 $$ \begin{aligned}   u_{1} (x,y,t) &  = e^{{i\omega ^{*} t}} \sum\limits_{{m = 1}}^{\infty } {\sum\limits_{{n = 1}}^{\infty } {U_{{mn}}^{{(1)}} \cos \left[ {\frac{{n\pi }}{a}\left( {x + \frac{a}{2}} \right)} \right]} } \sin \left[ {\frac{{m\pi }}{b}\left( {y + \frac{b}{2}} \right)} \right] \\    v_{1} (x,y,t) &  = e^{{i\omega ^{*} t}} \sum\limits_{{m = 1}}^{\infty } {\sum\limits_{{n = 1}}^{\infty } {V_{{mn}}^{{(1)}} \sin \left[ {\frac{{n\pi }}{a}\left( {x + \frac{a}{2}} \right)} \right]\cos \left[ {\frac{{m\pi }}{b}\left( {y + \frac{b}{2}} \right)} \right]} }  \\    u_{3} (x,y,t) &  = e^{{i\omega ^{*} t}} \sum\limits_{{m = 1}}^{\infty } {\sum\limits_{{n = 1}}^{\infty } {U_{{mn}}^{{(3)}} \cos \left[ {\frac{{n\pi }}{a}\left( {x + \frac{a}{2}} \right)} \right]} \sin \left[ {\frac{{m\pi }}{b}\left( {y + \frac{b}{2}} \right)} \right]}  \\    v_{3} (x,y,t) &  = e^{{i\omega ^{*} t}} \sum\limits_{{m = 1}}^{\infty } {\sum\limits_{{n = 1}}^{\infty } {V_{{mn}}^{{(3)}} \sin \left[ {\frac{{n\pi }}{a}\left( {x + \frac{a}{2}} \right)} \right]\cos \left[ {\frac{{m\pi }}{b}\left( {y + \frac{b}{2}} \right)} \right]} }  \\    w(x,y,t) &  = e^{{i\omega ^{*} t}} \sum\limits_{{m = 1}}^{\infty } {\sum\limits_{{n = 1}}^{\infty } {W_{{mn}} \sin \left[ {\frac{{n\pi }}{a}\left( {x + \frac{a}{2}} \right)} \right]} } \sin \left[ {\frac{{m\pi }}{b}\left( {y + \frac{b}{2}} \right)} \right] \\  \end{aligned}  $$

where $U_{mn}^{(1)} ,     V_{mn}^{(1)} ,     U_{mn}^{(3)} ,   V_{mn}^{(3)} ,   W_{mn}^{{}}$ are coefficients in displacement function.

The equations of motion are acquired in accordance with the Rayleigh–Ritz method^34^.

13 $$ \frac{{\partial (U - T)}}{{\partial U_{{mn}}^{{(1)}} }} = 0,\;\frac{{\partial \left( {U - T} \right)}}{{\partial U_{{mn}}^{{(3)}} }} = 0,\;\frac{{\partial (U - T)}}{{\partial V_{{mn}}^{{(1)}} }} = 0,\;\frac{{\partial \left( {U - T} \right)}}{{\partial V_{{mn}}^{{(3)}} }} = 0,\;\frac{{\partial \left( {U - T} \right)}}{{\partial W_{{mn}} }} = 0 $$

By substituting Eqs. (9–12) into Eq. (13), we can simplify the characteristic equation and express it in matrix form as follows:

14 $$ \left\{ {\left[ {\begin{array}{*{20}c}    {K_{{11}} } &  \cdots  & {K_{{15}} }  \\     \vdots  &  \ddots  &  \vdots   \\    {K_{{51}} } &  \cdots  & {K_{{55}} }  \\   \end{array} } \right] - \left( {\omega ^{*} } \right)^{2} \left[ {\begin{array}{*{20}c}    {m_{{11}} } &  \cdots  & {m_{{15}} }  \\     \vdots  &  \ddots  &  \vdots   \\    {m_{{51}} } &  \cdots  & {m_{{55}} }  \\   \end{array} } \right]} \right\}\left[ X \right] = 0 $$

where $[X] = \left[ {U_{{mn}}^{{(1)}} ,U_{{mn}}^{{(3)}} ,V_{{mn}}^{{(1)}} ,V_{{mn}}^{{(3)}} ,W} \right]^{T} ;$ the symbol [K] represents the stiffness matrix, while the symbol [M] denotes the mass matrix.

We can calculate the circular frequency and loss factor of the FGCSVP by applying the following equation.

15 $$\omega = \sqrt {{\text{Re}} (\omega^{*} )^{2}   }, \;  \eta = {\text{Im}} ((\omega^{*} )^{2} )/{\text{Re}} (\omega^{*} )^{2} $$

**Figure 2:**
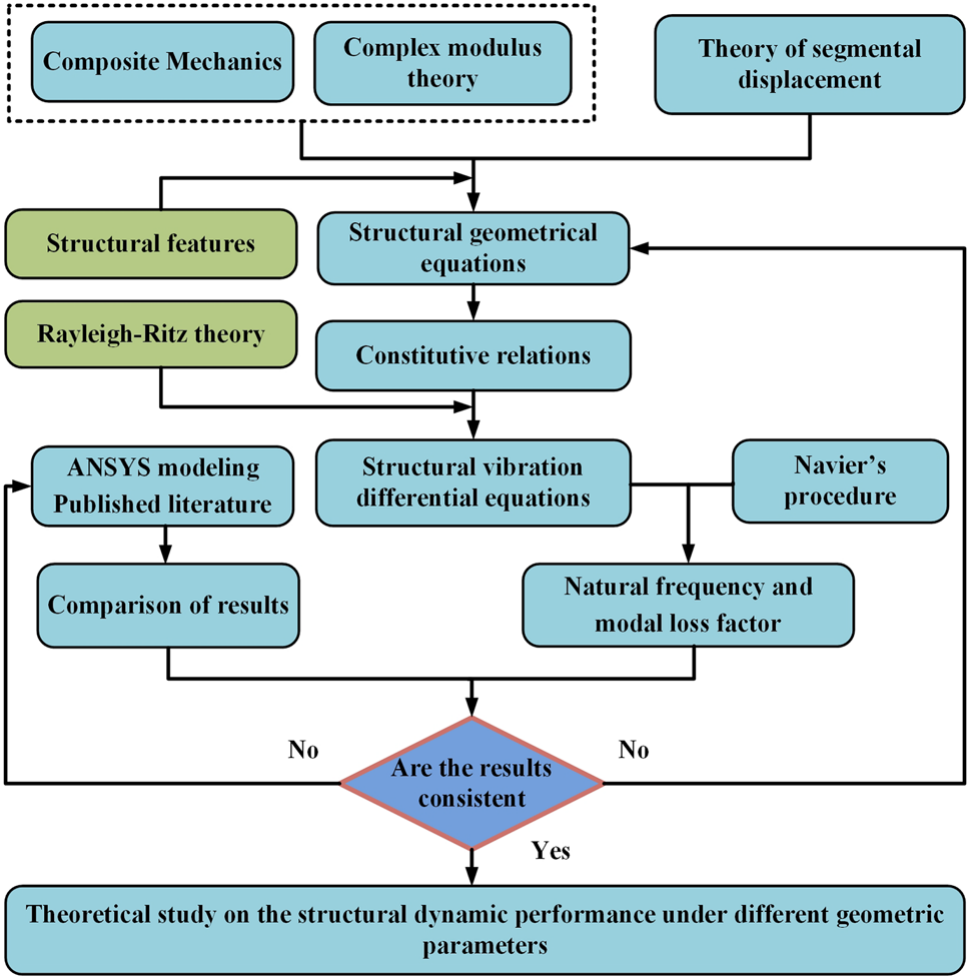
A flowchart for the theoretical approach.

A program will be developed to solve for the dynamic parameters of FGCSVP, based on Eqs. (1–15). By inputting the material and dimensional parameters of FGCSVP, the program will calculate the natural frequencies and loss factors. To validate the accuracy of the proposed theoretical model, the calculated results will be compared with published literature and ANSYS simulation results. Once the model is validated, it will be used to investigate the influence of different structural parameters on the dynamic performance of FGCSVP. Figure 2 presents a flowchart illustrating the theoretical approach.

## Model validation

To confirm the validity of the theoretical model and methods presented in this paper, two test configurations will be examined. Firstly, the non-dimensional dynamic parameters of the functionally graded composite structure will be calculated using Eqs. (1–15). Subsequently, the calculated results will be compared to the findings reported in published literature. Secondly, the ANSYS software is employed to compute the natural frequencies and loss factors of FGCSVP. Subsequently, the obtained results are juxtaposed with those derived from the theoretical method in the present study. By considering these test configurations and conducting the necessary comparisons, the accuracy and reliability of our proposed model and methods can be evaluated.

### First case

Firstly, we calculate the first through fifth natural frequencies of a functionally graded composite plate without embedded viscoelastic polymer using the theory described in this paper. In order to evaluate the accuracy of the computational results, a comparison is made between the results obtained from this paper and those obtained from reference^35^. The material parameters used in this study were adopted from the materials provided in reference^35^. The material parameters of ceramics are shown below: $\rho_{c}^{{}} = 3800 Kg/m^{3} ,$ $E_{c} = 380   GPa,$ and $\mu_{c} = 0.3$. The material parameters of metal are shown below: $\rho_{m}^{{}} = 2700 Kg/m^{3} ,$ $E_{m} = 70   GPa,$ and $\mu_{m} = 0.3$.

 Table 1 provides a comparison between the results obtained from the theoretical model developed in this study and the results reported in reference^35^. The non-dimensional frequency utilized in this section is denoted as $\Omega = \omega a^{2} \sqrt {\rho_{c} h/D_{c} }$, where *D*_*c*_ is flexural rigidity. Although the results in Table 1 closely resemble those presented in reference^35^, certain deviations can be observed. These deviations mainly stem from the differences in theoretical assumptions and constitutive models between the two theoretical frameworks. In reference^35^, the neglect of transverse shear deformation in the structure leads to a lower calculation accuracy compared to the theoretical model proposed in this paper.

**Table 1:** First through fifth non-dimensional frequency.

*a/b*	Sources	$\lambda_{1}$	$\lambda_{2}$	$\lambda_{3}$	$\lambda_{4}$	$\lambda_{5}$
0.2	Present	9.547	10.48	12.483	15.052	27.154
	Reference^28^	9.6035	10.712	12.626	15.374	26.678
0.5	Present	11.474	18.354	29.812	38.971	45.837
	Reference^28^	11.543	18.468	30.333	39.246	46.461
1	Present	18.354	45.837	45.837	73.264	91.518
	Reference^28^	18.468	46.171	46.171	74.288	93.723
2	Present	45.837	73.264	118.852	155.212	182.418
	Reference^28^	46.171	73.874	121.330	156.980	185.840

Next, the formula (1–15) of this paper is applied to calculate the first through fourth natural frequencies and loss factors of a functionally graded composite plate with viscoelastic polymer. Subsequently, the calculated results are compared with those in reference^33^. To maintain conciseness, the thickness ratio of each part from bottom to top is denoted by a combination of three numbers, such as "1-1-1" or "1-2-1". The material parameters used in this study were adopted from the materials provided in reference^33^. The non-dimensional frequency utilized in this section is denoted as $\Omega = \omega b^{2} /h\sqrt {\rho_{0} /E_{0} }$, $\rho_{0} = 1 kg/m^{3}$, $E_{0} = 1 GPa$.

 Tables 2 and 3 provides a comparison between the results obtained from the theoretical model developed in this study and the results reported in reference^33^. The remarkable agreement between the results obtained in this study for the non-dimensional dynamic parameters and those reported in the open literature is evident. The deviation observed between the two theoretical calculation results can be mainly attributed to the varying assumptions that were made and the differing descriptions of the material deformation behavior. In contrast to reference^33^, the controlled differential equations generated by this model contain only five unknown coefficients, representing a reduction of four unknown coefficients. Consequently, the theoretical model presented in this paper boasts higher computational efficiency compared to the theoretical model in reference^33^.

**Table 2:** First through fourth natural frequencies (*a/b* = 1, *a/h* = 0.05, *p* = *k* = 0.1).

Modal Numbers	Results	Non-dimensional frequency $\Omega$					
		1–1-1	1-2-1	1-8-1	2-1-2	2-1-1	2-2-1
1	Present	0.353	0.251	0.103	0.440	0.468	0.358
	Reference^33^	0.354	0.252	0.107	0.439	0.466	0.358
2	Present	0.874	0.615	0.216	1.088	1.161	0.885
	Reference^33^	0.873	0.616	0.218	1.085	1.155	0.883
3	Present	0.874	0.615	0.216	1.088	1.161	0.885
	Reference^33^	0.873	0.616	0.218	1.085	1.155	0.883
4	Present	1.393	0.978	0.325	1.737	1.854	1.412
	Reference^33^	1.390	0.979	0.329	1.727	1.836	1.406

**Table 3:** First through fourth loss factors (*a/b* = 1, *a/h* = 0.05, *p* = *k* = 0.1).

Modal Numbers	Results	Loss factors $\eta (\% )$					
		1-1-1	1-2-1	1-8-1	2-1-2	2-1-1	2-2-1
1	Present	3.75	6.94	48.72	3.07	2.41	3.49
	Reference^33^	3.75	6.93	48.43	3.08	2.42	3.50
2	Present	1.54	2.92	28.16	1.26	0.98	1.43
	Reference^33^	1.54	2.91	27.99	1.26	0.99	1.44
3	Present	1.54	2.92	28.16	1.26	0.98	1.43
	Reference^33^	1.54	2.91	27.99	1.26	0.99	1.44
4	Present	0.97	1.85	19.79	0.79	0.62	0.90
	Reference^33^	0.97	1.84	19.59	0.79	0.62	0.91

### Second case

By employing ANSYS, we were able to simulate the behavior of the proposed model under various conditions and compare the results with the theoretical predictions. However, due to the complexity of the FGCSVP, directly obtaining the loss factor using ANSYS software is not feasible. Instead, we employed the modal strain energy method^36,37^ to indirectly calculate the loss factor of the entire structure based on strain energy. In the finite element numerical simulation, the geometric model was divided into small elements, and the modal strain energy of each element was extracted. The strain energies of elements with the same material were then summed to obtain the strain energy of a specific material. By combining the strain energies of different materials, we obtained the strain energy of the entire structure. Equation (16) demonstrates the calculation of the modal loss factor for the entire structure. This factor is determined by dividing the dissipated energy of the viscoelastic polymer layer by the strain energy of the entire structure. Specifically, the strain energy of the viscoelastic polymers layer is multiplied by the loss coefficient of the viscoelastic polymer to obtain the dissipated energy of the rubber layer. The result is then divided by the strain energy of the whole structure. This approach ensures a more accurate representation of the dissipation characteristics and overall performance of the structure. Therefore, we utilized a combination of ANSYS software and the modal strain energy method to solve for the first four natural frequencies and the first four loss factors of the functionally graded composite damping structure, aiming to verify the accuracy of the theoretical model proposed in this paper.

16 $$ \eta^{r} = \frac{{\eta_{V} U_{V}^{r} }}{{U_{S}^{r} }} $$

where $\eta_{V}$ is the loss factor of viscoelastic polymer, $U_{V}^{r}$ is modal strain energy of viscoelastic polymer layer, and $U_{S}^{r}$ is the total modal strain energy.

For the ANSYS simulation, the SOLID 185 element type was utilized. The simulation model was divided into 80 elements in the length and width directions, and 6 elements in the thickness direction (2 elements were allocated for each upper and lower skin layer, and 2 elements for the viscoelastic polymer layer). The structural model adopted simple support boundary conditions along all four sides. The material parameters of the functionally graded composite layer are shown in "First case", while the material parameters of the viscoelastic polymer are shown below: $$\rho_{p}^{{}} = 999 Kg/m^{3} ,$$$$E_{p} = 2.684   MPa,$$$$ \mu_{p} = 0.498$$, and $$\eta_{{_{V} }}$$ = 0.9683. The corresponding calculation results are displayed in Table 4.

**Table 4:** Comparison of finite element calculation results with those of this paper (*a* = 1 m, *a/b* = 1, *h/a* = 0.05, *h*_*c*_ = *h*_*v*_ = *h*_*b*_, *p* = 0).

Modal numbers	Frequency (Hz)				Modal loss factor			
	First	Second	Third	Fourth	First	Second	Third	Fourth
ANSYS	74.630	186.89	186.89	292.35	0.0482	0.0194	0.0194	0.0126
Present	75.956	187.106	187.106	298.246	0.0466	0.0193	0.0193	0.0122
Error	1.78%	0.12%	0.12%	2.02%	− 3.32%	− 0.52%	− 0.53%	− 3.18%

As shown in Table 4, the errors between the natural frequencies and loss factors calculated by ANSYS and those obtained from the theoretical model developed in this paper are all within 4%. This confirms the validity of our theoretical model. Furthermore, we utilize this validated model to analyze the impact of the structure's size and material parameters on its dynamic performance.

## Results and discussions

In this section, we demonstrate the effects of graded index, damping layer position, aspect ratio, and layer thickness ratio on the first-order dimensionless natural frequency (FO-DNF) and first-order loss factor (FO-LF) of FGCSVP using a mutually verified model. Unless specified otherwise, the non-dimensional frequency used in this section is denoted as $\Omega = \omega b^{2} /h\sqrt {\rho_{0} /E_{0} }$, $\rho_{0} = 1 kg/m^{3}$, $E_{0} = 1 GPa$.

### Effects of the graded index of functional graded composite material layer on dynamic performance of FGCSVP

**Figure 3:**
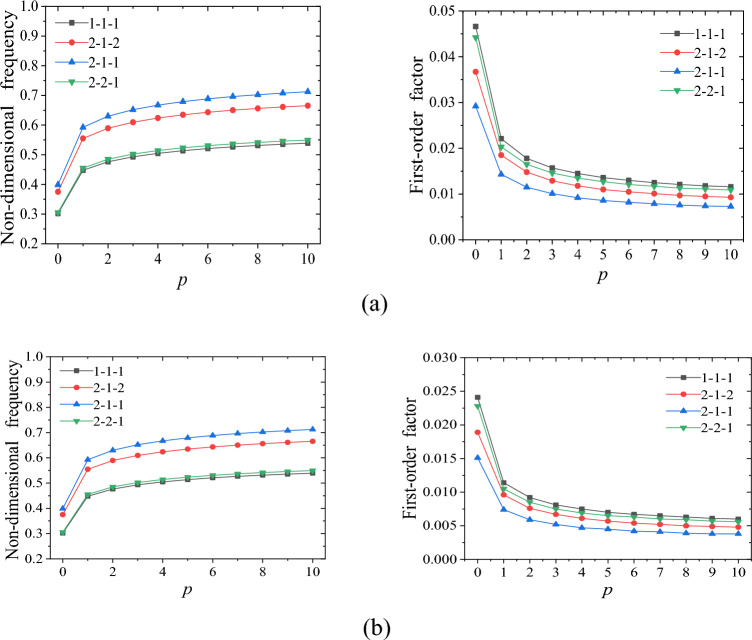
Impact of graded index on the dynamic performance of FGCSVP with varying structural parameters; (**a**) *h/a* = 0.05, *a/b* = 1, *h*_*1*_ = *h*_*3*_, *h*_*2*_/*h* = 1/3, η = 0.9683; (**b**) *h*/*a* = 0.05, *a/b* = 1, *h*_*1*_ = *h*_*3*_, *h*_*2*_/*h* = 1/3, η = 0.5.

The initial investigation focused on the impact of the graded index of the functional graded composite material layer on the structural properties. Figure 3a,b illustrates the effect of the graded index on the structural vibration characteristics, considering different thickness ratios of each layer. From Fig. 3a,b, it is evident that the FO-DNF of the structure rises with an increase in the graded index. However, when the graded index is large, the FO-DNF becomes less responsive to changes in the graded index. Conversely, the FO-LF of the FGCSVP decreases as the graded index increases. Moreover, as the graded index increases, the rate at which the FO-LF decreases slows down. This can be attributed to the fact that as the graded index increases, the proportion of ceramic materials in the entire structure also increases. Ceramic materials typically have a much higher elastic modulus than metal materials. Consequently, the increase in ceramic materials enhances the stiffness of the overall structure, thereby elevating the FO-DNF. However, the increase in ceramic materials leads to a decrease in its ability to dissipate energy during dynamic deformation.

Comparing Fig. 3a with Fig. 3b, we can see that the FO-LF of the structure in Fig. 3a is significantly higher than that in Fig. 3b, while there is no significant difference in the FO-DNF. This difference can be attributed to the higher loss factor of the viscoelastic damping material used in the structure depicted in Fig. 3a compared to that in Fig. 3b. As a result, the former has a greater capacity for dissipating energy during dynamic deformation. Moreover, we have noticed that the FO-LF of the structure with a thickness ratio of 1-1-1 is greater when compared to the structure with a thickness ratio of 2-2-1. Although the structure with a thickness ratio of 1-1-1 contains less viscoelastic polymer material than the 2-2-1 ratio, the arrangement of the viscoelastic polymer layer differs between the two structures, which contributes to this discrepancy. Therefore, the FO-LF of the former is greater than that of the latter. Next, we will investigate the impact of the viscoelastic polymer layer position on the vibration and damping characteristics of the structure.

### Effects of position of the damping layer on dynamic performance of FGCSVP

**Figure 4:**
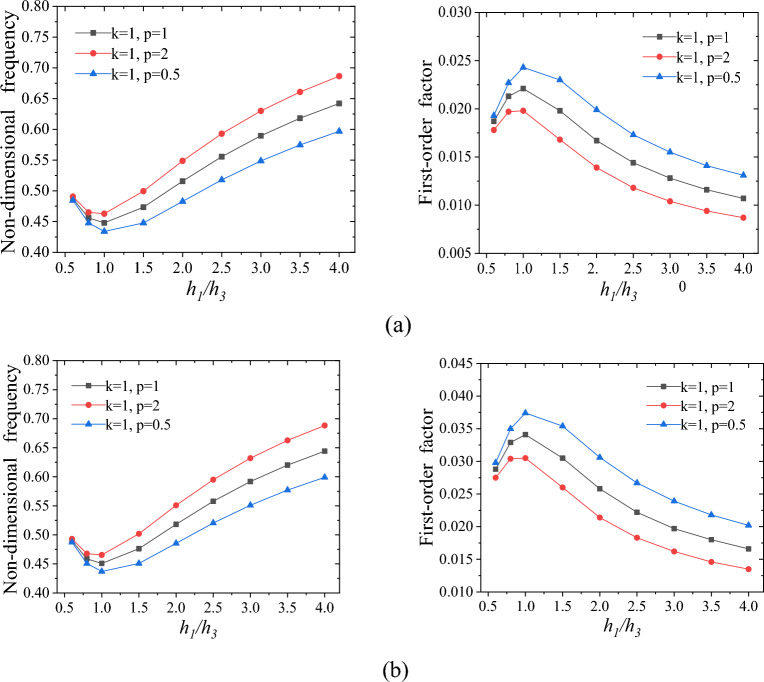
Impact of the values *h*_*1*_*/h*_*3*_ on the dynamic performance of FGCSVP with varying structural parameters; (**a**) *h/a* = 0.05, *a/b* = 1, *h*_*1*_ = *h*_*3*_, *h*_*2*_/*h* = 1/3; (**b**) *h*/*a* = 0.04, *a/b* = 1, *h*_*1*_ = *h*_*3*_, *h*_*2*_/*h* = 1/3.

In this section, we have examined how the position of the viscoelastic polymer layer affects the FO-DNF and the FO-LF of the FGCSVP. The impact of the viscoelastic polymer layer's position on these factors is depicted in Fig. 4. From Fig. 4, it is evident that when the ratio of *h*_*1*_ to *h*_*3*_ is 1, the structure exhibits the highest FO-LF and the lowest FO-DNF. This indicates that placing the viscoelastic polymer layer in the center of the plate maximizes the structure's ability to dissipate energy during dynamic deformation while minimizing its stiffness. Among the three different graded index structures illustrated in Fig. 4, the structure characterized by graded index values of *k* = 1 and *p* = 0.5 exhibits a higher FO-LF. Conversely, the structure with graded index values of *k* = 1 and *p* = 2 displays a higher FO-DNF. Further observation reveals that when the ratio of *h*_*1*_ to *h*_*3*_ is small, the FO-DNF and FO-LF of structures with three different graded indices are very close to each other. However, as the ratio of *h*_*1*_ to *h*_*3*_ increases, the differences in the FO-DNF and FO-LF of structures with three different graded indices gradually become more pronounced. This can be attributed to the fact that the graded index of the lower cortex is denoted by *p*, while the graded index of the upper cortex is denoted by *k*. As the ratio of *h*_*1*_ to *h*_*3*_ increases, the thickness of the lower panel layer with the graded index of *p* increases, while the thickness of the upper panel layer with the graded index of *k* decreases. In other words, the proportion of the thickness of the lower panel layer with different graded indexes in the overall structure also increases. Therefore, as the ratio of *h*_*1*_ to *h*_*3*_ increases, the differences in dynamic performance among the three structures with different graded indexes in the lower panel become more pronounced. Comparing Fig. 4a and Fig. 4b, it can be observed that when the value of *h/a* is smaller, the structure experiences a larger first-order loss factor.

### Effects of aspect ratio on dynamic performance of FGCSVP

**Figure 5:**
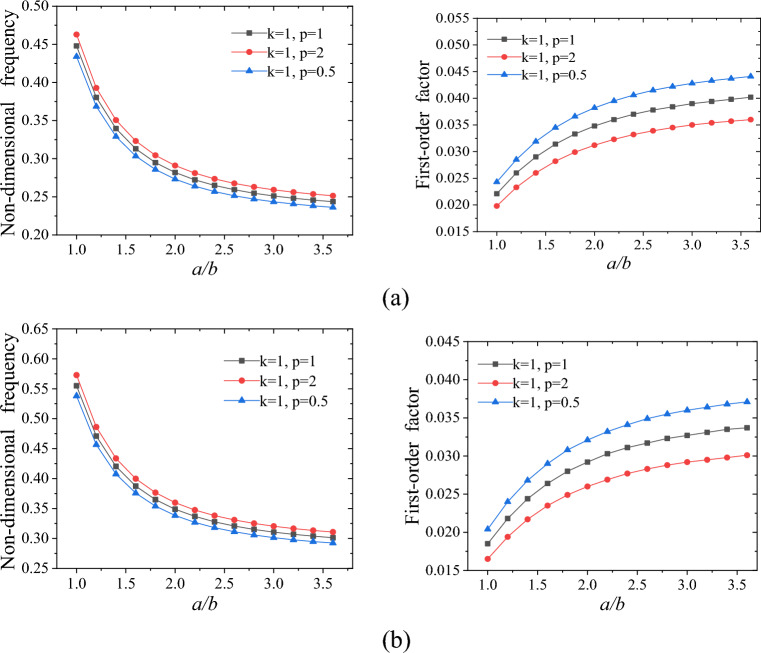
Impact of aspect ratio on the dynamic performance of FGCSVP with varying structural parameters; (**a**) *h/a* = 0.05, *h*_*1*_ = *h*_*3*_, *h*_*2*_/*h* = 1/3; (**b**) *h*/*a* = 0.05, *h*_*1*_ = *h*_*3*_, *h*_*2*_/*h* = 1/5.

Next, we investigate the impact of the value *a*/*b* on the FO-DNF and FO-LF of the structure. Figure 5 illustrates the influence of the value *a/b* on the FO-DNF and FO-LF of the FGCSVP. As shown in Fig. 5, the FO-DNF of the structure decreases as the value *a*/*b* increases. Specifically, when the value *a/b* is 3, the decline rate the FO-DNF is approximately 0. On the other hand, the FO-LF increases with an increase in the value *a/b*, although the rate of increase slows down as the value *a/b* increases. By comparing Fig. 5a and Fig. 5b, it can be observed that regardless of the value of *h*_*2*_*/h*, the dimensionless FO-DNF and FO-LF exhibit the same trend of change with respect to the value *a/b*.

### Effects of layer thickness ratio and elastic modulus on dynamic performance of FGCSVP

Finally, the vibration and damping characteristics of FGCSVP with different values *h*_*2*_*/h* and elastic modulus are studied. Table 5 illustrates the impact of *h*_*2*_/*h* values on the FO-DNF and the FO-LF. From Table 5, it is evident that as the value of *h*_*2*_/*h* increases, the FO-DNF of FGCSVP decreases, while the FO-LF increases. Additionally, as shown in Table 5, enhancing the elastic modulus of metal or ceramic materials leads to an increase in the FO-DNF and a decrease in the FO-LF of the FGCSVP.

**Table 5:** Impact of the values *h*_*2*_*/h* and elastic modulus on the dynamics parameters.

*h*_*2*_/*h*	*k* = 1, *p* = 1		*k* = 1, *p* = 2		*k* = 1, *p* = 0.5	
	$\Omega$	$\eta (\% )$	$\Omega$	$\eta (\% )$	$\Omega$	$\eta (\% )$
*E*_*m*_ = 70 GPa, *E*_*c*_ = 380 GPa						
0.2	0.5549	0.0185	0.5728	0.0165	0.538	0.0204
0.25	0.5144	0.019	0.5312	0.0169	0.4985	0.0209
0.3	0.4743	0.0205	0.49	0.0183	0.4596	0.0226
0.35	0.4348	0.0231	0.4492	0.0207	0.4211	0.0254
0.4	0.3956	0.0269	0.4089	0.0242	0.3831	0.0296
0.45	0.357	0.0325	0.3691	0.0292	0.3457	0.0356
0.5	0.3191	0.0404	0.3299	0.0365	0.3089	0.0443
*E*_*m*_ = 70 GPa, *E*_*c*_ = 400 GPa						
0.2	0.5648	0.018	0.584	0.0159	0.5468	0.0199
0.25	0.5236	0.0184	0.5415	0.0164	0.5068	0.0204
0.3	0.4828	0.0199	0.4995	0.0177	0.4672	0.022
0.35	0.4425	0.0224	0.4579	0.02	0.4281	0.0247
0.4	0.4027	0.0261	0.4168	0.0233	0.3894	0.0287
0.45	0.3634	0.0314	0.3762	0.0282	0.3513	0.0346
0.5	0.3247	0.0392	0.3362	0.0352	0.3139	0.0431
*E*_*m*_ = 90 GPa, *E*_*c*_ = 380 GPa						
0.2	0.5769	0.0167	0.5913	0.0151	0.5631	0.018
0.25	0.5348	0.0171	0.5483	0.0156	0.5218	0.0185
0.3	0.4932	0.0186	0.5058	0.0169	0.481	0.0201
0.35	0.452	0.0209	0.4637	0.0191	0.4407	0.0226
0.4	0.4112	0.0245	0.422	0.0223	0.4009	0.0264
0.45	0.371	0.0296	0.3808	0.027	0.3616	0.0319
0.5	0.3315	0.0369	0.3403	0.0338	0.3229	0.0399

This is mainly because the elastic modulus measures how a material responds to stress, indicating its ability to resist deformation under external load. A higher elastic modulus means a stronger resistance to deformation and results in a stiffer structure. By increasing the elastic modulus of ceramics or metals in functionally graded materials, the overall stiffness of the structure is indirectly enhanced, leading to a higher natural frequency. However, it is worth noting that increasing the elastic modulus of metals or ceramics also reduces their ability to dissipate energy during dynamic deformation.

## Conclusions

This study introduces a dynamic analysis model for FGCSVP, which has been validated by comparing the results with those obtained from finite element modal strain energy analysis and published literature. The model is then utilized to investigate the structural dynamic properties. The study also explores and illustrates the variations of FO-DNF and FO-LF. Based on the findings, the following conclusions can be drawn:

When the upper and lower skins are symmetrical, and the viscoelastic polymer layer is positioned within the neutral layer of the overall structure, the FGCSVP can achieve maximum FO-LF while minimizing FO-DNF.

Increasing the graded index of functional graded composite materials can enhance their FO-DNF and reduce the FO-LF of the FGCSVP. When the graded index is large, the FO-DNF and FO-LF of the FGCSVP become less sensitive to changes in the graded index of the material.

The FO-DNF of the FGCSVP decreases as the value of *a*/*b* increases. When a/b reaches 3, the decline rate of its FO-DNF is approximately 0. On the other hand, the FO-LF of the FGCSVP increases with the increase of a/b, but the rate of increase slows down as *a*/*b* increases.

## Data Availability

Data will be made available on request.

## References

[1] Li, Y. *et al.* A review on functionally graded materials and structures via additive manufacturing: From multi-scale design to versatile functional properties. *Adv. Mater. Technol.* **5**(6), 1900981 (2020). 10.1002/admt.201900981

[2] Nasr Esfahani, M., Hashemian, M. & Aghadavoudi, F. The vibration study of a sandwich conical shell with a saturated FGP core. *Sci. Rep.* **12**(1), 4950 (2022). 35322107 10.1038/s41598-022-09043-w PMC8942987

[3] Thongchom, C. *et al.* An analytical study of sound transmission loss of functionally graded sandwich cylindrical nanoshell integrated with piezoelectric layers. *Sci. Rep.* **12**(1), 3048 (2022). 35197511 10.1038/s41598-022-06905-1 PMC8866426

[4] Zhang, C. *et al.* Additive manufacturing of functionally graded materials: A review. *Mater. Sci. Eng. A* **764**, 138209 (2019). 10.1016/j.msea.2019.138209

[5] Ren, L. *et al.* Graded biological materials and additive manufacturing technologies for producing bioinspired graded materials: An overview. *Compos. B Eng.* **242**, 110086 (2022). 10.1016/j.compositesb.2022.110086

[6] Sharma, A. *et al.* A new process for design and manufacture of tailor-made functionally graded composites through friction stir additive manufacturing. *J. Manuf. Process.* **26**, 122–130 (2017). 10.1016/j.jmapro.2017.02.007

[7] Punera, D. & Kant, T. A critical review of stress and vibration analyses of functionally graded shell structures. *Compos. Struct.* **210**, 787–809 (2019). 10.1016/j.compstruct.2018.11.084

[8] Rajan, T. P. D. & Pai, B. C. Developments in processing of functionally gradient metals and metal–ceramic composites: A review. *Acta Metallurg. Sin. (Engl. Lett.)* **27**, 825–838 (2014). 10.1007/s40195-014-0142-3

[9] Zhou, H. *et al.* The fabrication of functional gradient hypereutectic Al-Si composites by liquid-solid separation technology. *J. Alloy. Compd.* **763**, 49–55 (2018). 10.1016/j.jallcom.2018.05.204

[10] Chao, Z. L. *et al.* Microstructure and mechanical properties of B4C/2024Al functionally gradient composites. *Mater. Des.* **215**, 110449 (2022). 10.1016/j.matdes.2022.110449

[11] Ituarte, I. F. *et al.* Design and additive manufacture of functionally graded structures based on digital materials. *Addit. Manuf.* **30**, 100839 (2019).

[12] Chauhan, P. K. & Khan, S. Microstructural examination of aluminium-copper functionally graded material developed by powder metallurgy route. *Mater. Today Proc.* **25**, 833–837 (2020). 10.1016/j.matpr.2019.10.007

[13] Sain, M. K. *et al.* Fabrication and characterization of homogenous and functionally graded glass fiber reinforced polymer composites. *Mater. Today Proc.* **66**, 3602–3608 (2022). 10.1016/j.matpr.2022.07.115

[14] Put, S. *et al.* Advanced symmetrically graded ceramic and ceramic-metal composites. *J. Mater. Sci.* **39**, 881–888 (2004). 10.1023/B:JMSC.0000012917.51982.9d

[15] Singh, S. J. & Harsha, S. P. Nonlinear vibration analysis of sigmoid functionally graded sandwich plate with ceramic-FGM-metal layers. *J. Vibr. Eng. Technol.* **8**, 67–84 (2020). 10.1007/s42417-018-0058-8

[16] Zhao, J. L. *et al.* Vibration characteristics of functionally graded carbon nanotube-reinforced composite double-beams in thermal environments. *Steel Compos. Struct.* **43**(6), 797–808 (2022).

[17] Akgöz, B. & Civalek, Ö. Effects of thermal and shear deformation on vibration response of functionally graded thick composite microbeams. *Compos. Part B Eng.* **129**, 77–87 (2017). 10.1016/j.compositesb.2017.07.024

[18] Kim, N. I. & Lee, J. Geometrically nonlinear isogeometric analysis of functionally graded plates based on first-order shear deformation theory considering physical neutral surface. *Compos. Struct.* **153**, 804–814 (2016). 10.1016/j.compstruct.2016.07.002

[19] Atmane, H. A. *et al.* On the thermal buckling of simply supported rectangular plates made of a sigmoid functionally graded Al/Al 2 O 3 based material. *Mech. Solids* **51**, 177–187 (2016). 10.3103/S0025654416020059

[20] Raza, A., Talha, M. & Pathak, H. Influence of material uncertainty on vibration characteristics of higher-order cracked functionally gradient plates using xfem. *Int. J. Appl. Mech.* **13**(05), 2150062 (2021). 10.1142/S1758825121500629

[21] Benachour, A. *et al.* A four variable refined plate theory for free vibrations of functionally graded plates with arbitrary gradient. *Compos. Part B Eng.* **42**(6), 1386–1394 (2011). 10.1016/j.compositesb.2011.05.032

[22] Pandey, S. & Pradyumna, S. A layerwise finite element formulation for free vibration analysis of functionally graded sandwich shells. *Compos. Struct.* **133**, 438–450 (2015). 10.1016/j.compstruct.2015.07.087

[23] Kapuria, S., Bhattacharyya, M. & Kumar, A. N. Bending and free vibration response of layered functionally graded beams: A theoretical model and its experimental validation. *Compos. Struct.* **82**(3), 390–402 (2008). 10.1016/j.compstruct.2007.01.019

[24] Eghtesad, A., Shafiei, A. R. & Mahzoon, M. Study of dynamic behavior of ceramic–metal FGM under high velocity impact conditions using CSPM method. *Appl. Math. Modell.* **36**(6), 2724–2738 (2012). 10.1016/j.apm.2011.09.056

[25] Gardner, N., Wang, E. & Shukla, A. Performance of functionally graded sandwich composite beams under shock wave loading. *Compos. Struct.* **94**(5), 1755–1770 (2012). 10.1016/j.compstruct.2011.12.006

[26] Njim, E. K., Al-Waily, M. & Bakhy, S. H. A review of the recent research on the experimental tests of functionally graded sandwich panels. *J. Mech. Eng. Res. Dev.* **44**(3), 420–441 (2021).

[27] Peng, C. & Tran, P. Bioinspired functionally graded gyroid sandwich panel subjected to impulsive loadings. *Compos. Part B Eng.* **188**, 107773 (2020). 10.1016/j.compositesb.2020.107773

[28] Natarajan, S. & Manickam, G. Bending and vibration of functionally graded material sandwich plates using an accurate theory. *Finite Elem. Anal. Des.* **57**, 32–42 (2012). 10.1016/j.finel.2012.03.006

[29] Pandey, S. & Pradyumna, S. Analysis of functionally graded sandwich plates using a higher-order layerwise theory. *Compos. Part B Eng.* **153**, 325–336 (2018). 10.1016/j.compositesb.2018.08.121

[30] Frostig, Y., Birman, V. & Kardomateas, G. A. Non-linear wrinkling of a sandwich panel with functionally graded core—extended high-order approach. *Int. J. Solids Struct.* **148**, 122–139 (2018). 10.1016/j.ijsolstr.2018.02.023

[31] Wang, S. *et al.* Structural dynamic properties of stiffened composite plates with embedded multi-layered viscoelastic damping membranes. *Mech. Adv. Mater. Struct.* **20**, 1–14 (2022).

[32] Wang, S. *et al.* Free vibration of co-cured composite structures with different numbers of viscoelastic damping membranes. *Compos. Structu.* **247**, 112434 (2020). 10.1016/j.compstruct.2020.112434

[33] Yang, C. *et al.* A modified Fourier-Ritz solution for vibration and damping analysis of sandwich plates with viscoelastic and functionally graded materials. *Int. J. Mech. Sci.* **106**, 1–18 (2016). 10.1016/j.ijmecsci.2015.11.031

[34] Wang, S. *et al.* Bending properties of carbon fiber reinforced composite multilayer damping structures with different types of stiffeners. *Mech. Adv. Mater. Struct.* **20**, 1–17 (2023).

[35] Chakraverty, S. & Pradhan, K. K. Free vibration of functionally graded thin rectangular plates resting on Winkler elastic foundation with general boundary conditions using Rayleigh–Ritz method. *Int. J. Appl. Mech.* **6**(04), 1450043 (2014). 10.1142/S1758825114500434

[36] Ungar, E. E. & Kerwin, E. M. Jr. Loss factors of viscoelastic systems in terms of energy concepts. *J. Acoust. Soc. Am.* **34**(7), 954–957 (1962). 10.1121/1.1918227

[37] Zhang, S. H. & Chen, H. L. A study on the damping characteristics of laminated composites with integral viscoelastic layers. *Compos. Struct.* **74**(1), 63–69 (2006). 10.1016/j.compstruct.2005.03.008

